# Reversible oxidation of ethylene on ferroelectric BaTiO_3_(001): An X-ray photoelectron spectroscopy study

**DOI:** 10.1016/j.heliyon.2024.e35072

**Published:** 2024-07-23

**Authors:** Alexandru-Cristi Iancu, Adela Nicolaev, Nicoleta G. Apostol, Laura E. Abramiuc, Cristian M. Teodorescu

**Affiliations:** a National Institute of Materials Physics, Atomiștilor 405A, 077125, Măgurele, Ilfov, Romania; b University of Bucharest, Faculty of Physics, Atomiștilor 405, 077125, Măgurele, Ilfov, Romania

**Keywords:** Ethylene, Ferroelectric surfaces, Barium titanate, Surface reactivity, X-ray photoelectron spectroscopy (XPS)

## Abstract

Adsorption and desorption of ethylene on BaO-terminated (001) barium titanate are investigated by X-ray photoelectron spectroscopy. Carbon is found in an oxidized state, at a binding energy similar to that resulting from CO adsorption on BaTiO_3_(001). The amount of carbon adsorbed on the surface is also similar to the case of CO/BaTiO_3_(001). Upon heating the substrate up to the loss of its ferroelectric polarization, the C 1s signal from the oxidized spectral region vanishes. At the same time, there was no noticeable oxygen depletion of the surface after repeated C_2_H_4_ adsorption and desorption. The substrate remains stable after repeated oxidative adsorption and desorption of ethylene. Desorption occurs at different temperatures, depending on the adsorption temperature, which suggests different adsorption geometries: non-dissociated adsorption at high temperature with ethylene bond on two surface oxygen atoms, and locally dissociated adsorption at lower temperatures, in “formaldehyde-like” local configurations.

## Highlights

• Adsorption of zero dipole moment hydrocarbon molecule on an ultraclean ferroelectric.
• Ethylene, when adsorbed on (001) barium titanate, gets oxidized.
• Adsorbed species are desorbed by heating above the Curie temperature of the substrate.
• Adsorption governed by molecular polarization due to the ferroelectric substrate.
• Adsorption as ethylene bound to surface oxygen or formaldehyde configuration.
• The barium titanate substrate not affected by repeated adsorption/desorption cycles.

## Graphical abstract

**Figure:**
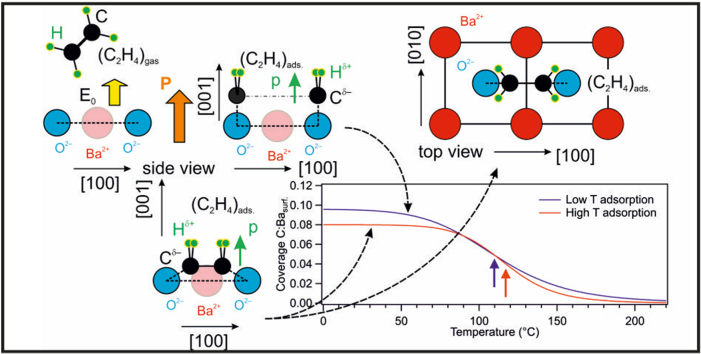

## 1 Introduction

Ferroelectric surfaces are investigated since almost two decades in view of their ability to foster molecular adsorptions, increase residence time and promote surface reactions [[1], [2], [3], [4], [5], [6], [7], [8], [9], [10], [11]]. Owing to the surface band bending, ferroelectric materials with out-of-plane polarization promote (photogenerated) carrier transport towards surfaces, rendering these surfaces oxidative or reductive [5,6,10,[12], [13], [14]]. A ferroelectric thin film, even with single domain structure, is stabilized by the formation of accumulated (compensating or stabilization) charges in its volume near surface or on adlayers (contaminants, metal contacts) [10]. Recently, a new theoretical view of these single domain films pointed out that charges accumulated at interfaces or near surfaces play a tantamount role in the stabilization of the ferroelectric state [15,16]. Nonetheless, the surface potential of a clean ferroelectric surface can be significant [17,18] and hence the interaction with gas phase molecules are expected to be important.

Not surprisingly, adsorption of polar molecules occurs naturally on ferroelectric surfaces [1,2,5,6,9,12,18]. It is easy to estimate that the electric field produced in vacuum by a sample with out-of-plane polarization of 1 C m^−2^ with a molecule whose dipole moment is 1 D yields to an interaction energy of about −2.35 eV when the dipole is aligned with the electric field of the substrate. As a consequence, even a molecule with small dipole moment, such as CO with 0.1 D experiences an interaction energy of about 47–71 meV, larger in magnitude than the thermal energy at room temperature, on barium titanate with an out-of-plane polarization of 0.2–0.3 C m^−2^. The interaction energy may become stronger if one takes into account the polarizability $\alpha =4\pi \epsilon_{0}\alpha_{v}$ of the molecule, where $\epsilon_{0}$ is the vacuum permittivity and $\alpha_{v}$ the polarizability in volume units [19]. As sketched in Ref. [20], even a molecule without an initial dipole moment in gas phase may become polarized in the neighborhood of the ferroelectric surface, and the interaction energy is about $-\alpha E^{2}/2\approx -2\pi \alpha_{v}P^{2}/\epsilon_{0}$, where $E\approx P/\epsilon_{0}$ is the electric field at the ferroelectric surface and P the polarization of the ferroelectric. A simple estimate for $P=1\;C∙m^{-2}$ and $\alpha_{v}=1\;{Å}^{3}$ gives an interaction energy of around 4.43 eV. Therefore, this molecular polarization by the field of the ferroelectric substrate may be more important than the interaction with the initial dipole moment. In the case of CO, with the polarizability $\alpha_{v}=1.95\;{Å}^{3}$ [19,20] and assuming $P=0.2\;C∙m^{-2}$, the interaction energy is expected to be on the order of 0.345 eV, several times higher than the interaction energy of the initial dipole moment with the electric field of the ferroelectric material. This process may offer a physical explanation of the “shallow physisorption well, dominated by van der Waals interactions and influenced by polarization” or “physisorption precursor state” introduced in Ref. [2] to explain the CO_2_ adsorption (with null dipole moment in the gas phase) on BaTiO_3_(001) (BTO(001) in the following). Moreover, the order of magnitude of the above interaction energy with the substrate and the quadratic dependence with the polarization explains well the dissociative adsorption of CO on Pb(Zr,Ti)O_3_(001) (PZT(001) in the following) [7] with a strong polarization (more than 1 C m^−2^) and the non-dissociative adsorption of CO on BTO(001) (polarization around 0.2–0.3 C m^−2^) [20]. To definitely evidence the latter mechanism, i. e. the interaction of the ferroelectric surface with a polarizable molecule, one needs to perform such kind of experiments for molecules with zero polarization in their ground state in gas phase. Methane, ethane, ethylene or acetylene are amongst the first candidates for such experiments, since they have null static dipole moment in their gas phase and polarizabilities $\alpha_{v}$ exceeding that of CO (2.59, 4.47, 4.25 and 3.93 Å^3^, respectively [19]). We stress that this kind of interaction is a completely new concept, since it was proposed for the first time this year, in Ref. [20].

In order to perform such experiments, the cleanness of the substrate is essential. The method used in Refs. [7,20] was X-ray photoelectron spectroscopy (XPS) using synchrotron radiation in surface sensitive regime, since this method is supposed (i) to quantify atomic compositions and amount of adsorbates; (ii) to reveal their chemical states; (iii) to assess the substrate polarization, *via* the surface band bending [[21], [22], [23]]. It is also important to quantify if the substrate is not affected by repeated adsorption and desorption processes. Indeed, in the case of CO/PZT(001) it was found that upon thermally induced desorption of reduced carbon on the surface, the substrate is depleted in oxygen [7]. The relative advantage of a strong polarization in enabling molecular dissociation on the surface was mitigated by the non-stability of the sample for repeated experiments. In the case of CO/BTO(001), it was found that the molecule does not dissociate on the surface, but it is gently desorbed at higher temperature, without affecting the stoichiometry of the surface [20].

As discussed above, in order to get more insight on this mechanism of molecular polarization induced by the electric field of the ferroelectric substrate, similar experiments should be performed starting with molecules with null dipole moment in the gas phase. In this work, we present the first experiment, pointing on the adsorption of ethylene (C_2_H_4_) on BTO(001) in conditions similar to that of Ref. [20]. The main surprise was that carbon is found in a similar state as for CO/BTO(001), with the C 1s spectrum featuring a single component. A toy model for the adsorption geometry was proposed and may be used as an input for *ab initio* computations. Thermally induced desorption was also followed-up by fast XPS and, by applying a Langmuir model, adsorption energies were derived. The stability of the surface stoichiometry upon repeated cycles of adsorption/desorption was also evidenced. Apart for the insights in fundamental aspects of molecular interactions with ferroelectric surfaces, successful adsorption and controlled desorption of hydrocarbons by ferroelectric surfaces, with the ferroelectric material unaffected by repeated adsorption-desorption cycles paves the way towards the use of such materials to mitigate the effect of greenhouse gases through carbon capture, utilization and storage (CCUS) technologies.

## 2 Experimental

The experiments were performed in the CoSMoS (combined spectroscopy and microscopy on surfaces) end station connected to the SuperESCA beamline at the Elettra (Trieste) synchrotron radiation facility, using horizontal linearly polarized soft X-rays. The analysis chamber operates in a base pressure of low 10^−10^ to 10^−11^ hPa. XPS was performed by using a Phoibos (Specs) 150 analyzer with angular acceptance of ±7°. Pass energies were 5 eV for C 1s and Ba 4d, measured with 390 eV photon energy, and 10 eV for survey spectra, Ti 2p, Ba 4d and O 1s, all measured with 650 eV photon energy, and again 5 eV for Ba 4d measured with 200 eV photon energy. The photoelectron take-off angle was 24°, and the angle between the direction of incoming X-rays and the detected photoelectrons was 90°, hence the detected electrons are in a direction parallel to the polarization of incoming soft X-rays. The estimated resolving power (combined broadening due to the beamline and to the analyzer) was better than 500, related to the photoelectron kinetic energy (≤0.2 eV instrumental broadening for ∼100 eV electron kinetic energy).

BaTiO_3_(001) (BTO) thin films were grown on 0.5 % Nb-doped SrTiO_3_(001) (STON) by pulsed laser deposition (PLD) in a Surface setup, using a KrF laser (248 nm wavelength) with repetition rate 5 Hz and laser fluence 1.5 J/cm^2^. The substrate was heated at 700 °C and the partial O_2_ pressure was 14 Pa. The 2” (diameter) × 0.125” (thickness) target was barium titanate provided by Praxair, with 99.9 % purity. After deposition, the sample was cooled down in a rich O_2_ atmosphere, 0.1 MPa, with a rate of 10 °C/min. X-ray diffraction revealed the tetragonal crystal structure of BTO(001) with lattice parameters *a* = 3.905 Å (in plane) and *c* = 4.121 Å (out-of-plane, i. e. along the [001] direction), and X-ray reflectivity measurements allowed one to determine the BTO(001) film thickness, close to 12.5 nm, see Ref. [20]. BTO was grown on STON since it is expected that ultrathin films will present a polarization oriented outwards [23].

BTO(001) experienced air exposure between its synthesis by PLD and its introduction in CoSMoS. Therefore, a cleaning procedure was defined well in advance before the synchrotron radiation experiments. It consists in heating the BTO up to about 1150 K for 2000 s in an oxygen pressure of 5 × 10^−7^ hPa, then cooling down in oxygen 2 × 10^−6^ hPa. XPS spectra revealed a low amount of reduced carbon on the surface. BTO(001) was also characterized by low energy electron diffraction (LEED), see Ref. [20]. After cleaning, samples were characterized by core level XPS, then C_2_H_4_ was adsorbed at different temperatures: 26 °C, 30 °C, – 60 °C and 19 °C. These temperatures were chosen for two practical reasons: (i) Firstly, for possible industrial applications, one needs to investigate whether a visible effect in the adsorption might be induced by lowering the temperatures, but not too much, such as these lower temperatures might be achieved by industrial refrigeration systems (without use of cryogenic liquids); (ii) Secondly, the sample manipulator is designed such that the temperature stabilization is a delicate balance between the cool nitrogen flow (starting from liquid nitrogen) through very thin pipes and sample heating with a filament. There was also the need to cool down the sample as fast as possible after each preparation (annealing). In order to prevent accidental contamination of the surface which might occur if one waits a too long time for the temperature stabilization, we decided to set the pumping speed and the filament power to several values, such as to explore temperatures industrially reachable around the room temperature, with the guarantee that these temperatures will be stable during the desired adsorption processes and subsequent measurements. The C_2_H_4_ dose was similar in all cases: 5 × 10^−6^ hPa during 15 min, i. e. about 3.4 kL. After each dose, the surface was characterized by core level XPS, then it was heated up to 300 ± 20 °C by following-up the signals from different core levels (Ba 4d, C 1s, Ti 2p, O 1s) using ultrafast XPS. The heating rate was 10 ± 1 °C/min. Only after the dosing at 30 °C the BTO(001) surface was then cooled down in O_2_ at 2 × 10^−6^ hPa during 5 min; after the other three dosings followed by desorptions, the surface was cooled down in ultrahigh vacuum. At the end of the thermally-induced desorption process, the surface was again characterized, to confirm its cleanness.

## 3 Results and discussions

### 3.1 Core level X-ray photoelectron spectroscopy

**Fig. 1:**
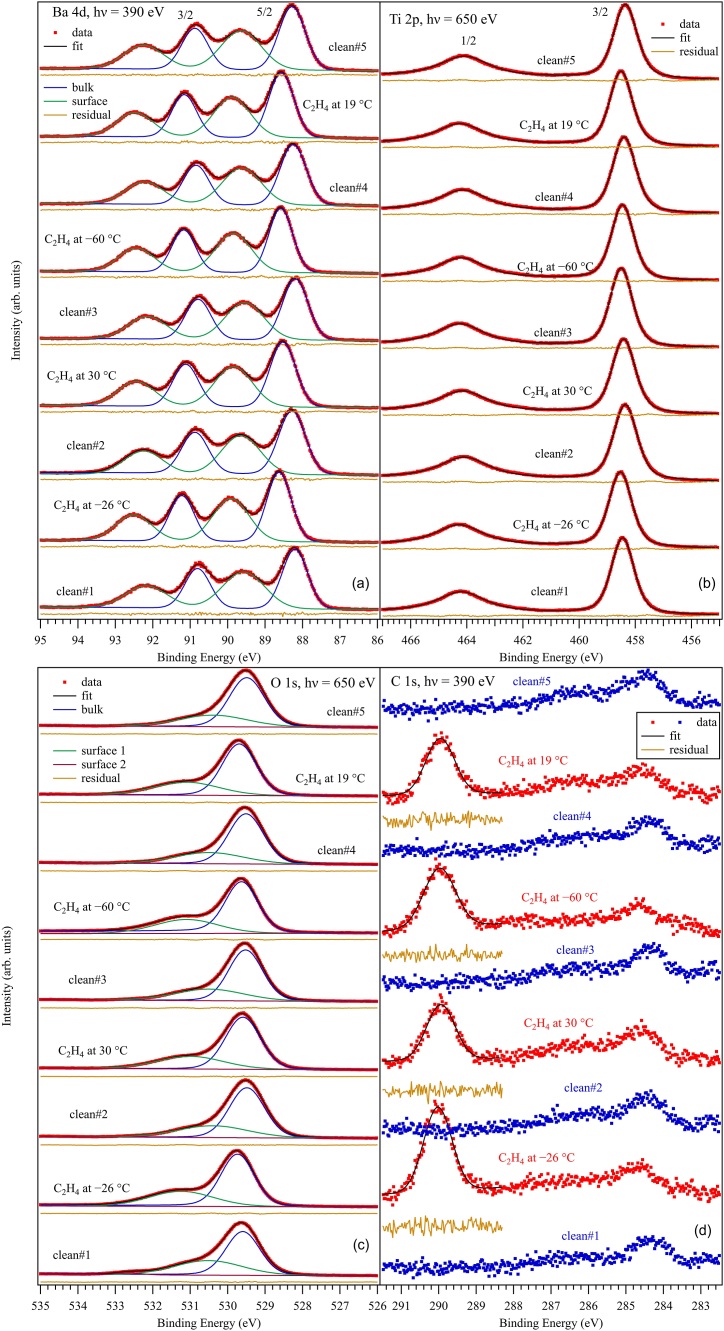
Core level X-ray photoelectron spectroscopy for Ba 3d (a), Ti 2p (b), O 1s (c) and C 1s (d) for BaTiO_3_(001) clean and after several dosing with 3.4 kL C_2_H_4_ at different temperatures. The employed photon energies are specified on each graph. Ba 4d spectra are simulated with two gaussian doublets plus inelastic backgrounds, Ti 2p spectra with a Voigt doublet, O 1s spectra with two Voigt lines plus backgrounds, and the C 1s spectra (only in the region above 288.5 eV) with one gaussian line plus background (black line).

Survey spectra are represented in the Electronic Supplementary Information, Fig. S1. Core level XPS spectra with enough statistics and density to allow ‘deconvolutions’ for BTO(001) after several adsorption and desorption processes are represented in Fig. 1 (Ba 4d and C 1s excited with 390 eV photon energy, Ti 2p and O 1s excited with 650 eV). Fig. S2 represents Ba 4d spectra obtained with 200 eV and 650 eV. The spectra should be ‘deconvoluted’ with Voigt profiles, which are convolutions of lorentzian and gaussian lineshapes. The lorentzian character is linked to the core level width of the investigated level, and the gaussian width is ascribed to the experimental broadening due to the monochromator and to the electron energy analyzer. However, since the Ba 4d and C 1s core level widths are considerably lower than the experimental broadening, these levels are simulated with gaussian lineshapes only. The Ti 2p and O 1s lines are simulated by using an analytical approximation of the Voigt profile (or pseudo-Voigt) [24]. To these lines, separate backgrounds are added which are primitives of the gaussian function (error function) for C 1s and Ba 4d or primitives of the pseudo-Voigt lines, computed also in Ref. [24], for Ti 2p and O 1s. The reason for associating a separate background to each chemically shifted component is that, for surface components, it is expected that no inelastic scattering of emitted photoelectrons occur in their way out to the analyzer, hence the background coefficient should vanish [20,25]. In the following, we shall call a (chemically-shifted) ‘component’ a single line used in the simulation of singlets (C 1s and O 1s) or a spin-orbit split doublet used in the simulation of Ba 4d and Ti 2p.

By comparing the spectra from Fig. 1(a) with those from Fig. S2, it is clear that the higher energy component of Ba 4d has a surface nature, since its relative weight increases drastically when one decreases the photon energy and thus the kinetic energy of the electrons, by exploring the more surface sensitive region. Also, the inelastic background associated to this component is nearly zero. In the following, we will call ‘Ba1’ the low binding energy (BE) component, ascribed to bulk and ‘Ba2’ the high energy component, ascribed to surface Ba. Relevant fitting parameters are nearly the same for all spectra: gaussian widths 0.900 ± 0.030 eV for Ba1 and 1.205 ± 0.062 eV for Ba2, spin-orbit splitting 2.597 ± 0.002 eV for Ba1 and 2.598 ± 0.003 eV for Ba2, and branching ratios 1.536 ± 0.014 for Ba1 and 1.651 ± 0.012 for Ba2. Binding energies and amplitudes (in fact, composition ratios obtained from these amplitudes) feature relevant differences for the investigated surfaces and will be commented more extensively below.

The Ti 2p spectra may be simulated well with a single doublet, therefore there is no surface component of Ti. This is an additional proof that the sample is BaO terminated. The core level (lorentzian) widths for the two lines of the Ti 2p doublet are quite different (0.454 ± 0.028 eV for 2p_3/2_ and 1.860 ± 0.052 eV for 2p_1/2_), a fact which is well known in XPS of Ti 2p and is due to the considerably lower core hole lifetime of the 2p_1/2_ level with respect to the 2p_3/2_ one, owing to the opening of additional Coster-Kronig decay channel for the former [26]. The gaussian width was 0.703 ± 0.010 eV. What is less known and was revealed the first time in Ref. [20] is that the branching ratio between the line areas of the 2p_3/2_ and 2p_1/2_ levels deviates considerably from its theoretical value of 2, it is 1.435 ± 0.019 for Ti 2p in BaO-terminated BTO(001). In Ref. [20] this finding was extensively discussed and a sketch of an explanation was provided, pointing on the important scattering of emitted photoelectrons from Ti 2p levels with higher angular momenta by the oxygen anions sitting on top of bulk Ti cations. Finally, the spin-orbit splitting was found at 5.741 ± 0.003 eV. The binding energies and amplitudes will be commented below.

The O 1s spectra present two components, a lower energy one denoted by ‘O1’ which is related to bulk BTO(001) and a higher energy one, ‘O2’, related to surface BaO. When CO was adsorbed on BTO, a third component at even higher BE had to be introduce to simulate well the spectra. In the actual case, no such component was needed. Moreover, the O 1s ‘deconvolution’ for C_2_H_4_/BTO(001) was performed by allowing three Voigt lines in the simulation, and systematically the amplitude of the third component went to zero. Therefore, it seems that upon C_2_H_4_ adsorption, oxygen is in a similar state as for clean BTO(001), up to band bending variations (see below). Relevant parameters: lorentzian width 0.117 ± 0.034 eV, gaussian widths 1.018 ± 0.045 eV for O1 and 2.054 ± 0.091 eV for O2, pointing on larger disorder for surface oxygen.

**Fig. 2:**
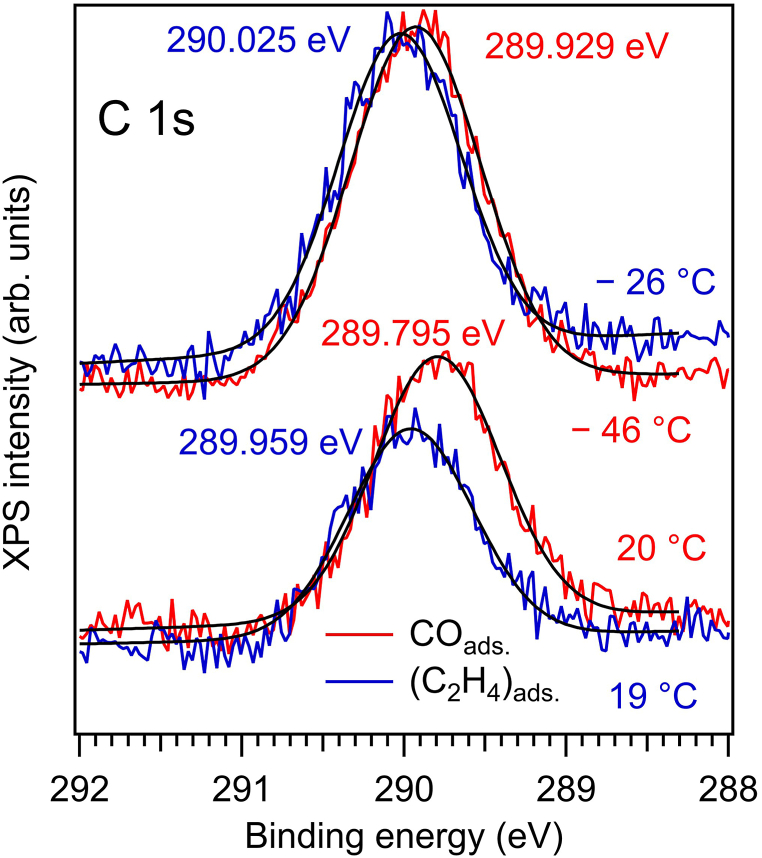
C 1s spectra obtained at 390 eV for CO and C_2_H_4_ adsorbed on BaTiO_3_(001) at nearly room temperature and at lower temperature. The peak energies are indicated according to the ‘deconvolution’ with a gaussian lineshape, black lines for all four spectra. The data for CO/BaTiO_3_(001) are the same as those from Ref. [20].

The C 1s main feature occurs in a region quite close to the case of CO/BTO(001). Fig. 2 presents comparisons between C 1s spectra for CO/BTO(001) and C_2_H_4_/BTO(001). The calibration to the Fermi level and that of the monochromator were identical in the two cases. It seems that for ethylene, the C 1s spectrum is shifted by about 0.1 eV towards higher BEs. This shift can be due to a stronger interaction of carbon atoms with oxygens on the surface, but also can be ascribed to band bending effects. The (purely gaussian) width of the C 1s line was estimated at 0.92 ± 0.05 eV.

One may advance the hypothesis that no C_2_H_4_ from the dosed gas is adsorbed on the BTO(001) surface, but instead CO from the residual gas is responsible for the observed spectral feature occurring in the C 1s spectrum. For that reason, we conducted a test experiment with time-resolved acquisition of all XPS spectra along a time roughly double to the dosing time (35 min. vs. 15 mins.). The substrate temperature during this experiment was 12 °C. No noticeable change in all core levels was observed. In Fig. S3 we present C 1s spectra obtained immediately after cleaning the BTO(001) crystal, after 35 min of continuous measurement, compared with the C 1s spectrum obtained after dosing ethylene at −26 °C. Clearly, in this test experiment there is no C–O peak occurring in the C 1s spectrum. The other possibility would be to have the ethylene from the laboratory gas bottle contaminated by CO, CO_2_ (etc.) but, in order to exceed the background vacuum by one order of magnitude for the partial pressure of impurities, these impurities should be in the range of 1000 ppm, which is unlikely. Also, we use this ethylene gas bottle for many experiments of graphene growth on reactive clean metal surfaces and no O 1s or C–O related peaks were observed in any of these experiments [[27], [28], [29]].

**Fig. 3:**
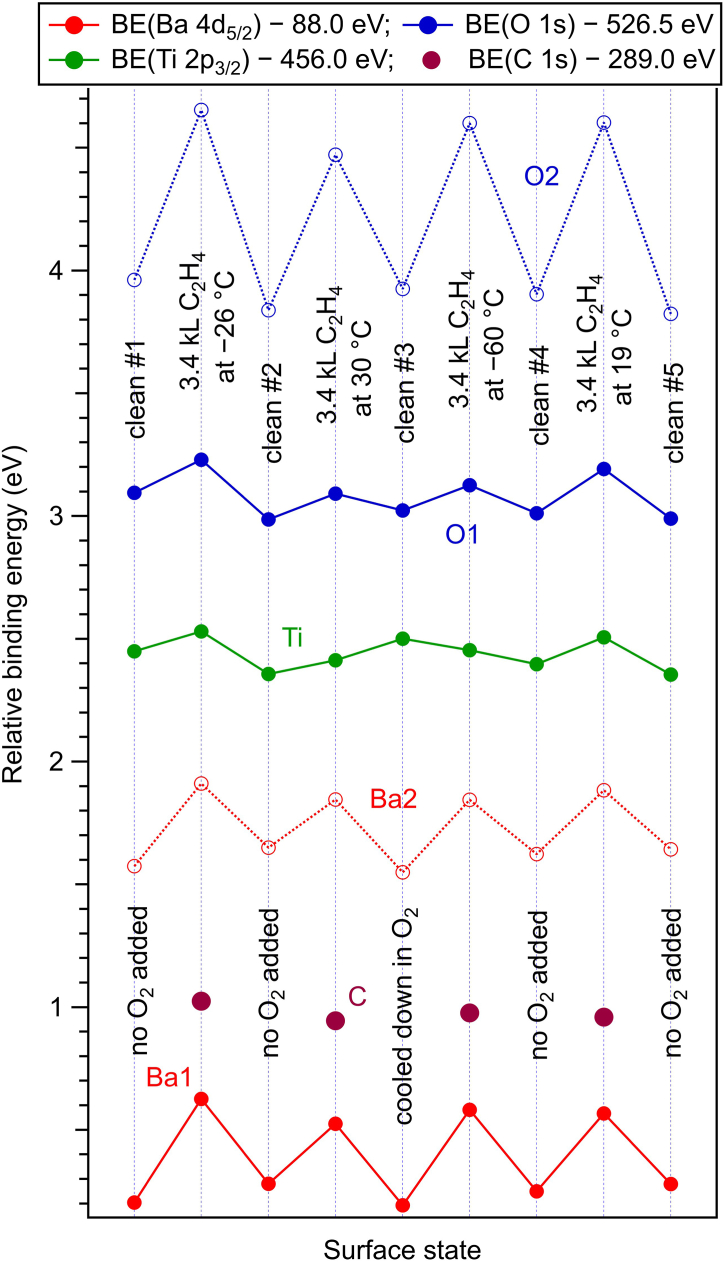
Relative binding energies from substrate atoms and for the different ‘components’, for several surface states (different clean surfaces, C_2_H_4_ dosing at different temperatures).

Fig. 3 presents an overview of the BEs of all components used in the deconvolution, for several surface states, by observing the history of the experiment. The most striking feature is the systematic increase in BE, especially for Ba and O, upon C_2_H_4_ adsorption. We anticipate that the BTO(001) exhibit a downwards band bending near surface, corresponding to a polarization oriented outwards [20,23]; this will become evident when one will analyze the temperature dependence of the XPS spectra up to the loss of the ferroelectric polarization. The variation is about 0.8 eV for surface O2, about 0.35 eV for surface Ba2, about 0.4 eV for bulk Ba1, and in the range of 0.1–0.2 eV for bulk O1 and for Ti. While a systematic increase of the BE for all core levels investigated can be attributed to an increase in magnitude of the band bending towards the surface of BTO(001), it could also point to an increasing interaction with surface oxygen atoms, which are responsible for the O2 component. Upon ethylene adsorption, surface oxygen seems to become less negative, as if some electrons would be transferred from the surface towards C_2_H_4_. The presence of ethylene, even in a finite amount (we anticipate that the higher amount of C:Ba2 ratios are in the range of 0.1) offers a new degree of freedom in stabilizing surface charges and strengthening the ferroelectric polarization [15]. It seems also that Ba cations (from bulk, i.e. Ba1) are more sensitive to the band bending than oxygen anions or Ti cations from the bulk. This could also mean that the stabilization (negative) charge was concentrated initially on those cations situated a few layers under the surface; when some more subjects for hosting this stabilization charge are available, namely the adsorbed ethylene, then the stabilization charge is distributed over these two possible hosts.

**Fig. 4:**
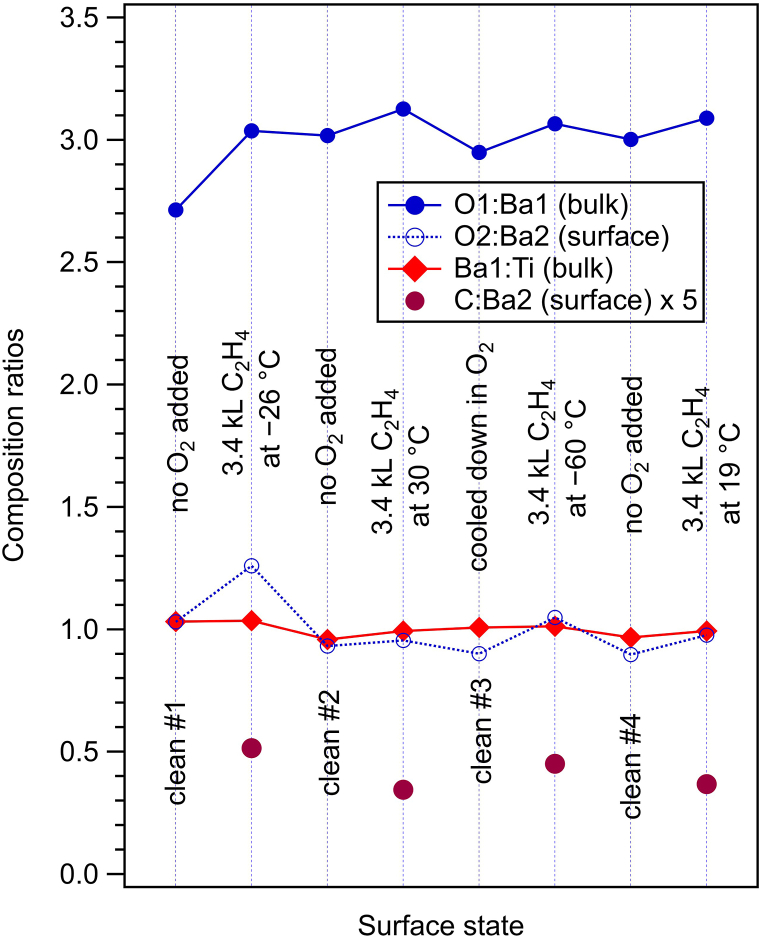
Corrected composition ratios obtained from the core level analysis. O1:Ba1 ratios are related to the bulk components, O2:Ba2 are related to surface components, Ba1:Ti are related to bulk components, and C:Ba2 are related to surface components.

The amplitude ratios obtained from the XPS data represented in Fig. 1 and S2, corrected by the cross sections and by the asymmetry parameters (see Ref. [20]) are far from a ‘perfect’ stoichiometry. Even if one corrects from the electron loss from Ti 2p, manifested in the low branching ratio r, by introducing a correction factor of c=3/(1+r)≈1.23 and one accepts that f≈0.33 of the O2 component still belongs to the bulk (see Ref. [20] for more details), one obtains (1−f)O2:Ba2≈0.98, (O1+fO2):Ba1≈1.87 and Ba1:(cTi)≈1.43, therefore the approximate stoichiometry would be Ba_1.43_TiO_1.87_. Apart from photoelectron diffraction effects, inelastic mean free path (IMFP) effects are very important for these spectra. In Ref. [20], a sophisticated Monte-Carlo simulation was developed in order to derive IMFPs for Ti 2p, O 1s and Ba 4d. It turned out that for IMFPs of 2.8, 3.0 and 5.9 Å for Ti 2p, O 1s and Ba 4d, respectively, a perfect stoichiometric film could yield amplitude ratios similar to those listed above. In the following, we shall just discuss ‘renormalized’ amplitude ratios with respect to their average values over all spectra analyzed, and multiplied with the ‘ideal’ stoichiometries (3 for O:Ba, 1 for Ba:Ti). These ratios are represented in Fig. 4. Except for the two ‘glitches’ in O1:Ba1 for the first clean surface and in O2:Ba2 for the first C_2_H_4_ dosing, which can be attributed to some beam variations on the sample, the composition ratios are rather stable, especially towards the end of the experiment. Consequently, within the experimental errors, we may infer that no noticeable oxygen depletion of the surface occurs after repeated adsorption/desorption processes. In particular, even the slight oxygen addition intended after the second dosing and desorption did not produce a noticeable enhancement of the oxygen content in the bulk nor in the surface component. As in the case of CO/BTO(001), desorption processes do not affect the BTO(001) surface.

**Fig. 5:**
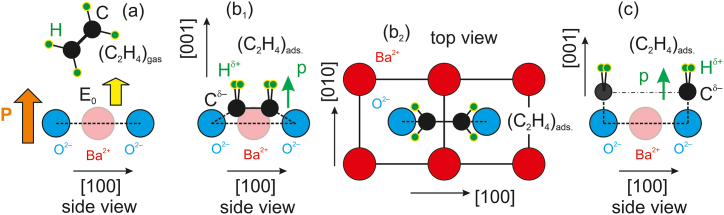
A simple adsorption model for ethylene on BaTiO_3_(001) which could explain the XPS observations, namely the position of the C 1s line, its single component character and the absence of any adsorbate-induced chemical shift in the O 1s line. (a) represents the situation (side view) prior to C_2_H_4_ adsorption. (b_1,2_) represents side and top views of the adsorption geometries supposing that the ethylene is not dissociated and just the C–C bond is weaken, allowing C–O bonds with adjacent oxygen ions from the first BaO layer. (c) a hypothetical situation with ethylene dissociated at the surface, adsorbed on top of surface oxygen, ‘formaldehyde-like’ configuration.

With these facts in mind, we may propose a model for the C_2_H_4_/BTO(001) adsorption. It is well known the fact that XPS is unable to detect hydrogen, but the fact that upon heating the sample the molecules are removed from the surface imply that hydrogen is still bond to carbon, otherwise the carbon – oxygen bonds would become too strong and carbon should be desorbed together with some oxygen from the surface, as for CO/PZT(001) from Ref. [7]. The second aspect is the fact that C 1s is well described by a single component, thus both carbon atoms should sit in similar environments. In Fig. 5(b) we present a sketch of the proposed adsorption geometry, by considering that the C=C double bond whose length is around 1.34 Å becomes a C–C single bond (1.53 Å) and considering also a distance of 1.42 Å for C–O bonds [30]. The distance between surface oxygen atoms is the same as the in-plane lattice parameter of BTO, found by X-ray diffraction to be a≈3.905Å [20]. There is no need to suppose any displacement of surface oxygen anions in order to accommodate the above bond lengths. As we specified in the Introduction, this model may be used as a starting point for more sophisticated *ab initio* computations. In Fig. 5(c) we present another hypothesis, where ethylene is dissociated and CH_2_ is adsorbed on surface oxygens, forming locally a ‘formaldehyde-like’ (H_2_C=O) configuration. This model has also to be taken into account when analyzing desorption data in the next Subsection.

The amount of carbon adsorbed, reflected by the C:Ba2 ratio, is similar for CO/BTO and for C_2_H_4_/BTO and is 9.5 ± 0.5 %, obtained when the adsorption takes place at low temperature. For adsorption near room temperature, this amount is around 7.1 ± 0.2 %.

### 3.2 Thermally induced desorption

**Fig. 6:**
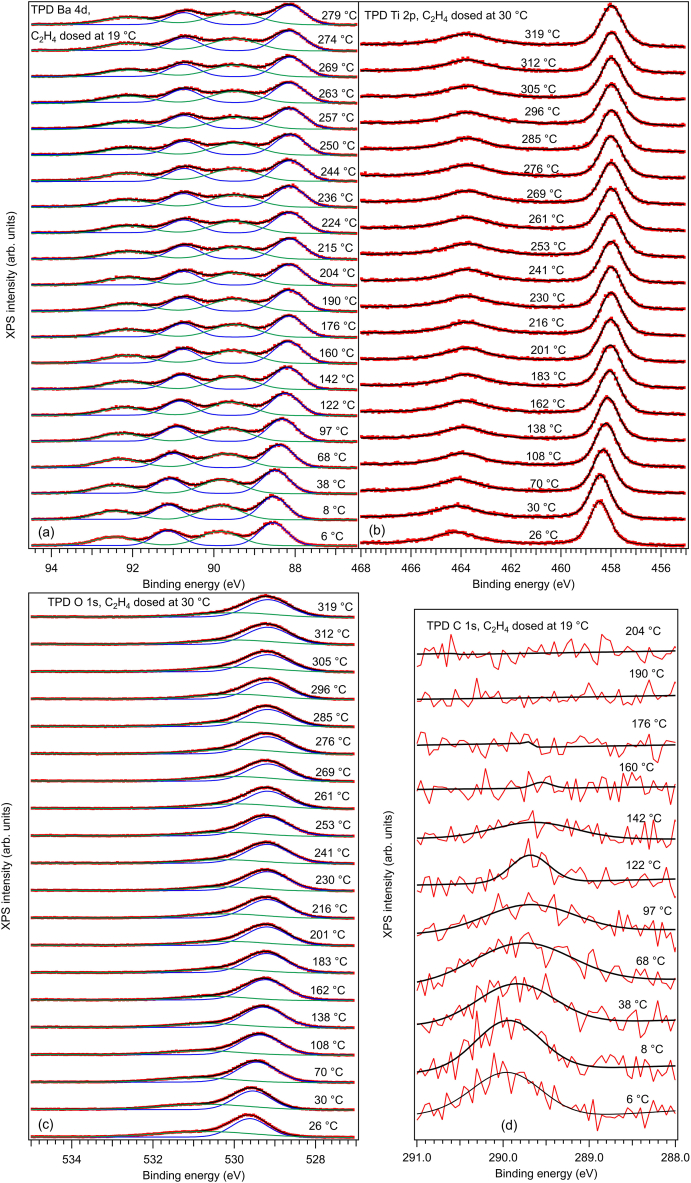
Temperature-programmed desorption of C_2_H_4_ initially adsorbed at near room temperature (3.4 kL), follow-up using all core levels: (a) Ba 4d; (b) Ti 2p; (c) O 1s; (d) C 1s. Dots are experimental data (red lines for C 1s). Full lines are fitting functions (black for the total fit) and separate contribution of two ‘components’ (for Ba 4d and O 1s).

**Fig. 7:**
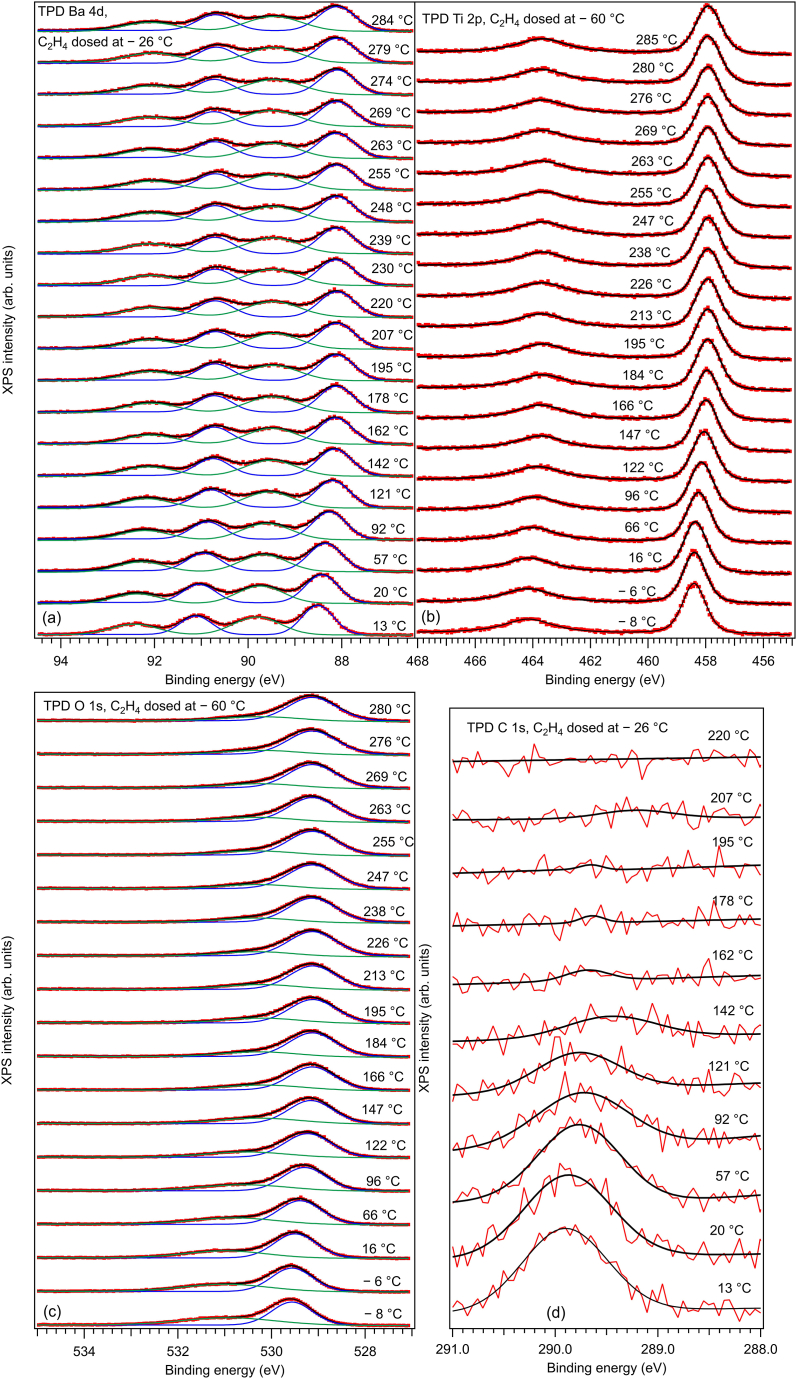
Same as the previous Figure, but for C_2_H_4_ initially adsorbed at low temperature.

**Fig. 8:**
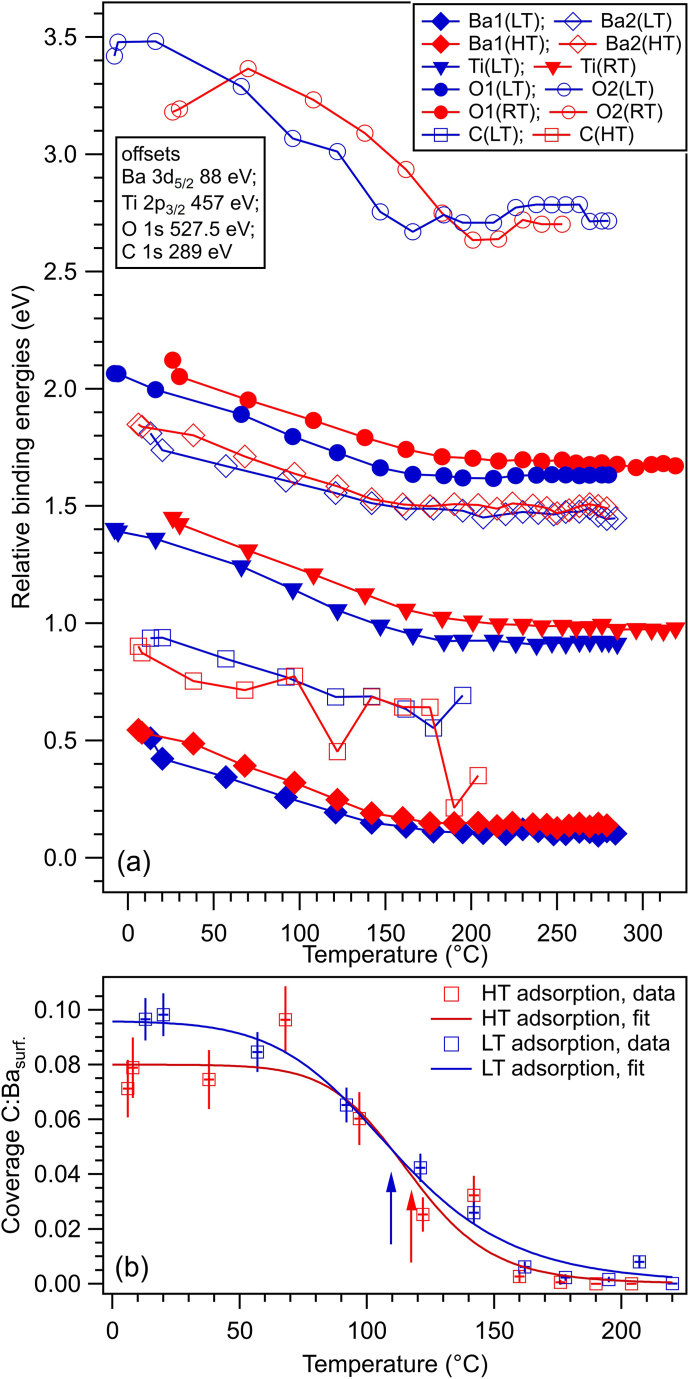
(a) Temperature evolution of binding energies of several ‘components’ and core levels, for 3.4 kL dosed at low temperature (blue symbols) and at higher temperature (red symbols). Error bars are on the order of the size of the symbols. (b) Temperature evolution of the carbon coverage obtained from the C 1s peak integral intensity, after dosing C_2_H_4_ at high (19 °C) or low (−26 °C) temperature. The fits are temperature dependence of coverage according to a simple Langmuir model, with constant adsorption energy. Arrows indicate the temperature where the coverage is half of its maximum value (red arrow for HT adsorption, blue arrow for LT adsorption).

The Curie temperature in bulk BTO is about 123 °C [31], but it might be expected to be higher by some tens of degrees in ultrathin films. Therefore, the BTO(001) after dosing was heated well above 200 °C, by taking advantage also on the previous experiment with CO/BTO(001). Fig. 6 represents the thermally programmed desorption (TPD) follow-up by fast XPS for C_2_H_4_/BTO(001) adsorbed near room temperature, while Fig. 7 represents the case of C_2_H_4_/BTO(001) adsorbed at low temperature. These fast XPS spectra were ‘deconvoluted’ with the same procedure as for the core level spectra from Fig. 1 and S2. Fig. 8 represents a summary of the evolution of binding energies and the C:Ba2 ratios.

It is clear that upon heating all core levels are moving towards lower BEs by a similar amount (0.44 ± 0.02 eV), except for the O2 component whose variation is about 0.77 ± 0.04 eV (Fig. 8(a)). This is a clear sign of disappearance of the band bending near the surface of the BTO(001). At the same time, the C 1s signal decreases drastically (Fig. 8(b)). As in all previous similar experiments, the relation of adsorbed carbon to the ferroelectricity of the substrate is obvious [7,10,20,32]. Next, the decrease of the C 1s intensity was simulated with a Langmuir function, such as [7,20]:

(1) $$\theta \left(T\right)=\frac{\theta_{0}}{1+\frac{k_{B}T{\left(2\pi Mk_{B}T\right)}^{3/2}}{ph^{3}}\exp\left(-\frac{W}{k_{B}T}\right)}$$

where θ(T) is the coverage at a given temperature T, $\theta_{0}$ the saturation coverage, $k_{B}$ the Boltzmann constant, h the Planck constant, M the molecular mass W the adsorption energy and p a parameter with dimension of pressure. The practical formula given by Eq. (18) from Ref. [20] may be used also in this case, since the mass of ethylene is the same as that of carbon monoxide. The fits are represented in Fig. 8(b) with full lines. The resulting fitting parameters are represented in Table 1.

**Table 1:** Parameters resulting from fits of the temperature dependence of the carbon coverage from Fig. 8(b), for low temperature and high temperature adsorption.

Parameter	Low Temperature	High Temperature
$\theta_{0}$ (related to Ba)	0.096	0.080
p[Pa]	1.34 × 10^6^	481 (170^a^)
W[eV]	0.451	0.727

a Using the mass 14 a. m.u. instead of 28 a. m. u. in eq. (1).

Note that with the polarizability $\alpha_{v}\left(C_{2}H_{2}\right)\approx 4.254\;{Å}^{3}$ [19] and a polarization of BTO(001) $P\approx 0.2\;C∙m^{-2}$, one obtains an energy $2\pi \alpha_{v}P^{2}/\epsilon_{0}\approx 0.754\;\text{eV}$, close to the adsorption energy value obtained for adsorption at high temperature. By using the polarizability of formaldehyde $\alpha_{v}\left(H_{2}\text{CO}\right)\approx 2.45\;{Å}^{3}$ and the same polarization of BTO(001) one obtains $2\pi \alpha_{v}P^{2}/\epsilon_{0}\approx 0.435\;\text{eV}$, close to the adsorption energy value obtained at low temperature. Hence, one may advance the hypothesis that at high temperature a configuration such as that from Fig. 5(b) is realized, with undissociated ethylene bond to two oxygen anions from the surface, while by adsorption at low temperature the ‘formaldehyde-like’ configuration sketched in Fig. 5(c) is possible. Irrespective on the adsorption model which needs validation by other theoretical and experimental methods (e. g. vibrational spectroscopy), the net result suggested by Fig. 8(b) is that carbon is desorbed at different temperatures following the adsorption at high or low temperature. One may speculate that at lower temperature the polarization of barium titanate could be larger and this might induce dissociation of ethylene molecules prior to their adsorption on the surface. By using the formula for the interaction energy proposed in Ref. [20] $\left|W_{i}\right|\left[\text{eV}\right]\approx 4.43\alpha_{v}\left[{Å}^{3}\right]P^{2}\left[C∙m^{-2}\right]$, it is easy to derive that a polarization $P\approx 0.63\;C∙m^{-2}$ could be sufficient to break the C=C bond in ethylene (7.545 eV dissociation energy [33]). Since the above formula includes a factor 1/2 coming from $\left|W_{i}\right|=\int p\left(E\right)dE=\int \alpha EdE=\alpha E^{2}/2$, where α is the polarizability, E the electric field experienced by the molecule and p(E) its dipole moment, it is easy to derive that if one supposes simply $\left|W_{i}\right|=p\left(E\right)E=\alpha E^{2}$, the polarization needed to break the C=C bond of ethylene becomes on the order of $0.45\;C∙m^{-2}$.

If the above hypothesis concerning the different adsorption geometries (Fig. 5(b and c)) holds, the fragments emitted by the desorption following ethylene adsorption at different temperatures should be different. Through heating the substrate following the adsorption at low temperature, one should expect that ethylene is released in vacuum, whereas while heating after adsorption at high temperature, smaller fragments might be ejected. Similar findings were reported for methanol adsorption on BTO, where there is a distinct peak of formaldehyde ejection [2]. Despite having installed a quadrupole mass analyzer (QMA) in the analysis chamber, we were not able to quantify desorption peaks, and a brief estimate of orders of magnitude follows. The sample surface in our experiment was 0.25 cm^2^, therefore the total number of adsorbed carbon atoms can be estimated as about 1.7 × 10^13^. Assuming that the desorption occurs in about 10 min, corresponding to a temperature rise to 200 °C and the pumping speed is 500 l/s, the total volume pumped during the desorption was 300 m^3^ and this yields to a density of desorbed species of 5.5 × 10^10^ m^−3^. By using the Boltzmann equation, the partial pressure yields about 2.2 × 10^−10^ Pa, far below the detection limit. Experiments are planned for the future with reduction by at least two orders of magnitude the pumping speed during the desorption, using a considerably smaller dedicated vessel to mitigate desorption from the walls, and by using samples of at least 4 cm^2^ surface and arranging the pumping such as the flow passes through the QMA. This might give an overall factor of about 200, rendering the pressure increase due to desorption observable with commercial QMAs.

## 4 Conclusions

The successful adsorption of a non-polar molecule (ethylene) on a ferroelectric surface (BaO terminated BaTiO_3_(001)) reveals some further aspects to be investigated. Although X-ray photoelectron spectroscopy was once more proven to be a highly useful technique to quantify adsorbates and their chemical states, together with the interplay between the coverage of adsorbed molecules and the substrate polarization (analyzed through the surface band bending at free ferroelectric surfaces), open questions arose regarding the adsorption geometries and the nature of the thermally desorbed species. Adsorption geometries may be investigated through X-ray photoelectron diffraction (XPD) and/or surface sensitive vibrational spectroscopy (IR absorption or Raman spectroscopy), and validated *via ab initio* computations. The nature of desorbed species may be investigated in a dedicated mass spectroscopy experiment, as described in the last paragraph of the last Subsection. These investigations are planned for the future. However, the complete XPS experiment and the adsorption models presented in Fig. 5 might be used as a reasonable starting point for the foreseen experiments or computations.

The results presented in this work also open the avenue for further experiments on adsorption of non-polar molecules (especially of methane or carbon dioxide) on ferroelectric substrates, which could have interesting applications for direct air capture plants expected to be built during the last years to reduce greenhouse gas content in our planet's atmosphere [34]. Quite recently, our group obtained similar results in CO_2_ adsorption and desorption from BaTiO_3_(001). CO_2_ also has null dipole moment in gas phase, considerable polarizability (2.91 Å^3^), and is the main responsible for the greenhouse effect yielding the global warming. The success in the CO_2_ adsorption on BaTiO_3_(001) reinforces the proposed mechanism stemming in the electric field provided by the clean ferroelectric surface towards the vacuum and its effect in polarizing incoming molecules, enabling their attachment on the surface. These results will be presented in another work. Similar experiments on adsorption of methane, ethane and acetylene are also planned.

## Data availability statement

All data and resulting analyses will be made available on request.

## Ethics declarations

All authors have made substantial contributions to the following: (1) the conception and design of the study, the acquisition of data, or the analysis and interpretation of data; (2) drafting the article or critically revising its important intellectual content; (3) final approval of the version submitted. All authors and responsible authorities where the work was carried out have approved this publication. All authors disclose any financial or other interests related to the submitted work that (1) could affect or have the perception of affecting the author's objectivity, or (2) could influence or have the perception of influencing the content of the article. All authors and their immediate family members disclose any personal financial interests (e. g. stocks or shares in companies with interests related to the submitted work or consulting fees from companies that could have interests related to the work), professional affiliations, advisory positions, board memberships, or patent holdings that are related to the subject matter of this contribution.

## CRediT authorship contribution statement

**Alexandru-Cristi Iancu:** Visualization, Validation, Resources, Methodology, Investigation, Data curation. **Adela Nicolaev:** Visualization, Methodology, Investigation, Data curation. **Nicoleta G. Apostol:** Supervision, Methodology, Investigation, Formal analysis, Data curation. **Laura E. Abramiuc:** Visualization, Validation, Formal analysis, Data curation. **Cristian M. Teodorescu:** Writing – review & editing, Writing – original draft, Visualization, Validation, Supervision, Software, Resources, Project administration, Methodology, Investigation, Funding acquisition, Formal analysis, Data curation, Conceptualization.

## Declaration of competing interest

The authors declare that they have no known competing financial interests or personal relationships that could have appeared to influence the work reported in this paper.
