# Supplementary material for: Reversible oxidation of ethylene on ferroelectric BaTiO3(001): An X-ray photoelectron spectroscopy study
Source: Heliyon. 2024 Jul 23;10(15):e35072. doi: 10.1016/j.heliyon.2024.e35072 (PMC11328086; doi:10.1016/j.heliyon.2024.e35072)
Supplement: Multimedia component 1 [file mmc1.docx]

Electronic Supplementary Information

Reversible oxidation of ethylene on ferroelectric BaTiO_3_(001): an X-ray photoelectron spectroscopy study

Alexandru-Cristi Iancu,^1,2^ Adela Nicolaev,^1^ Nicoleta G. Apostol,^1^ Laura E. Abramiuc,^1^

and Cristian M. Teodorescu^1,^*

^1^National Institute of Materials Physics, Atomiștilor 405A, 077125 Măgurele, Ilfov, Romania

^2^University of Bucharest, Faculty of Physics, Atomiștilor 405, 077125 Măgurele, Ilfov, Romania

*corresponding author, e-mail [teodorescu@infim.ro](mailto:teodorescu@infim.ro)


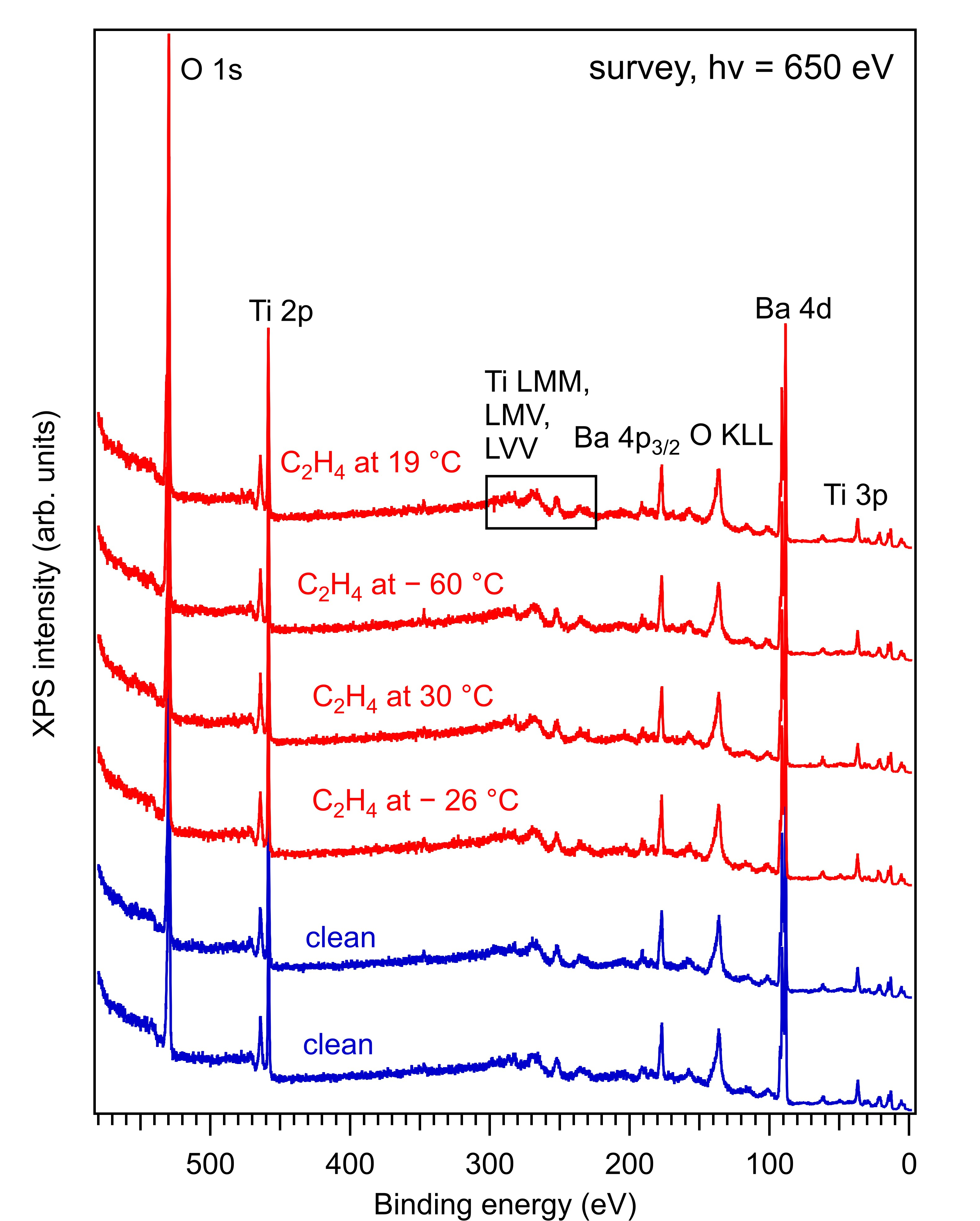


Figure S1. Survey spectra obtained at photon energy 650 eV for two preparations of BaTiO_3_(001) (blue curves) and for ethylene adsorbed at different temperatures. Note that the C 1s signal is not visible in these survey spectra, owing to its low photoionization cross section.


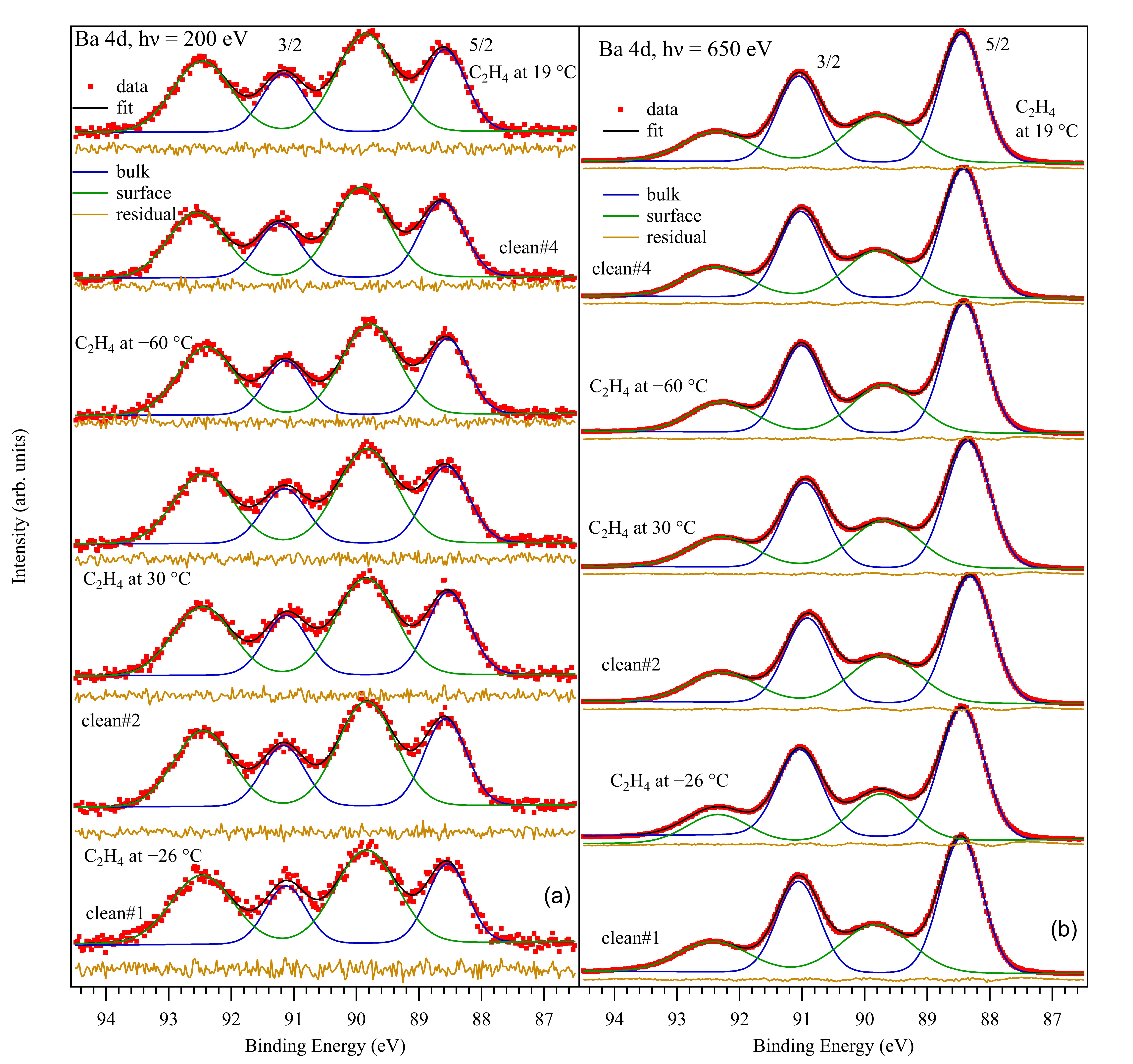


Figure S2. Ba 4d core level spectra obtained at two different photon energies. Ba 4d spectra are simulated with two gaussian doublets plus inelastic backgrounds, as in Fig. 1.


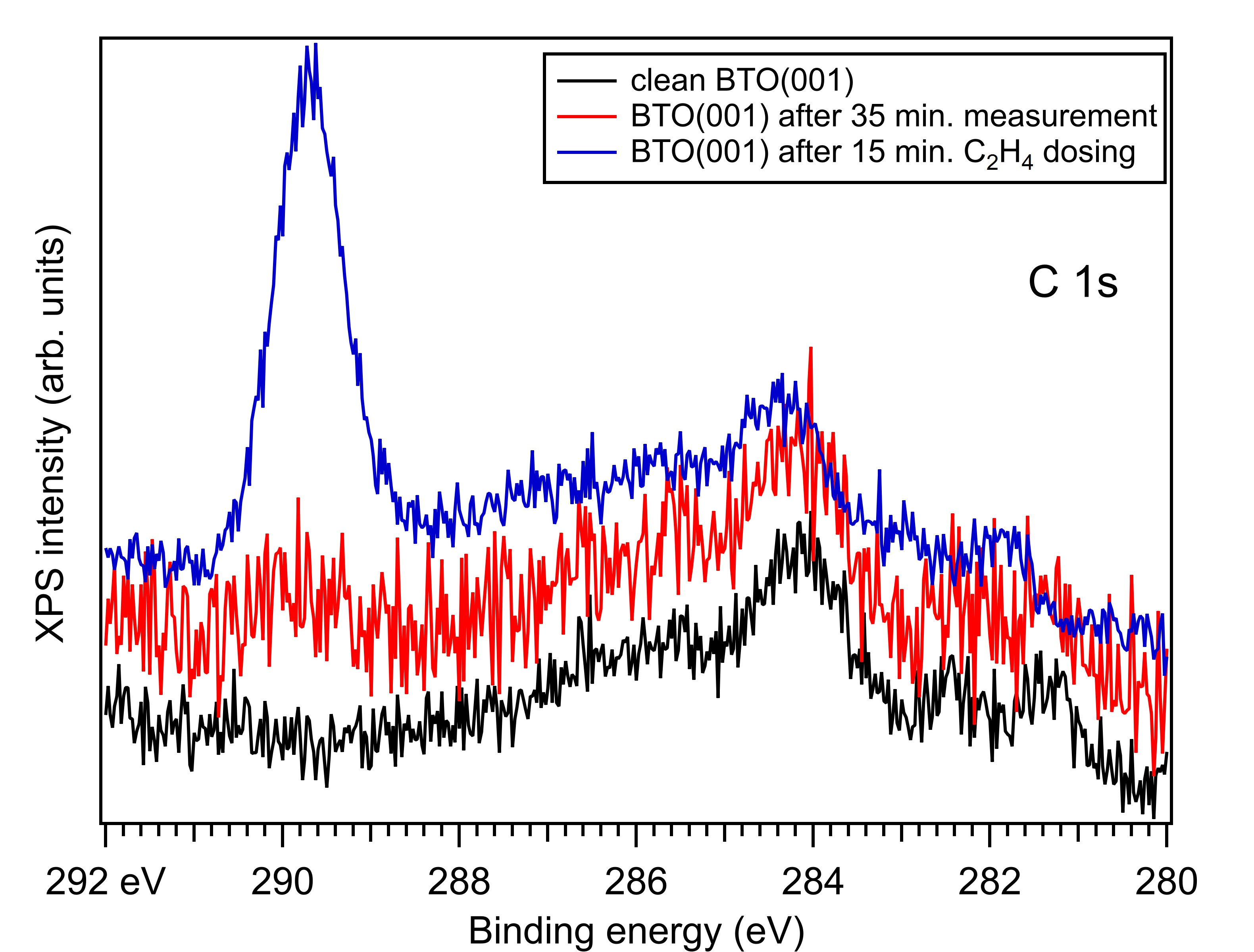


Figure S3. C 1s spectra obtained on clean BaTiO_3_(001) (black), after 35 minutes of continuous measurement in ultrahigh vacuum (2 × 10^–10^ hPa) (red) at 12 °C and after 15 minutes of C_2_H_4_ dosing at 5 × 10^–6^ hPa, –26 °C.
